# Papillary Microcarcinoma of Sublingual Thyroid Gland: A Case Report

**DOI:** 10.7759/cureus.6810

**Published:** 2020-01-29

**Authors:** Hani Z Marzouki, Ahmad Aldajani, Mazin Merdad, Wafa Saber, Ibrahim Amer

**Affiliations:** 1 Otolaryngology - Head and Neck Surgery, King Abdulaziz University, Jeddah, SAU; 2 Otolaryngology - Head and Neck Surgery, University of Jeddah, Jeddah, SAU; 3 Medicine, King Abdulaziz University, Jeddah, SAU; 4 Medicine, King Abdulaziz University Hospital, Jeddah, SAU

**Keywords:** ectopic thyroid, papillary microcarcinoma

## Abstract

Ectopic thyroid gland is a rare condition where the thyroid is not placed in the pre-tracheal region. Majority of cases are commonly located at the lingual portion. The malignant shift of ectopic thyroid is considered a rare manifestation with a challenging surgical approach. Here we report our management and surgical approach of a patient with papillary microcarcinoma of sublingual thyroid gland presented to our facility.

## Introduction

Ectopic thyroid (ET) gland is an uncommon condition where the thyroid is not located in the pre-tracheal region [1]. In embryonic life, the thyroid gland starts to migrate down from the base of the tongue through the thyroglossal duct until it reaches its standard location [2]. Ectopic thyroid is developed by descending arrest in thyroglossal tract [3]. The majority of cases are located at the lingual portion accounting for approximately 90% of cases [4]. Other sites include sublingual region, submandibular or anterior tongue, and it was also found in the trachea, larynx, mediastinum, and heart [4, 5].

The prevalence of ectopic thyroid is about 1:100,000-300,000, where it significantly rises to 1:4000-8000 in patients with functional thyroid disease; it is more common in females with a female to male ratio of 4:1. It could present at any age, from five months until the age of 40 or older. However, it is more common to present at a younger age group [5].

Ectopic thyroid can present in the form of goiter or mass. Due to its abnormal position, it can be liable for oropharyngeal obstruction, with a presentation of dyspnea or dysphagia [6]. On the other hand, ectopic thyroid might be found as an incidental finding [7]. In the absence of an orthotropic thyroid, most ectopic thyroids patients suffer from hypothyroidism, with only two cases that have been reported in the literature were hyperthyroidism [7-10].

All diseases that can affect the normal thyroid gland are capable of affecting the ectopic thyroid, such as inflammation, hyperplasia, adenoma, and malignancy. The incidence of malignant transformation in ectopic thyroid is equal to normally placed thyroid [11]. Most ectopic thyroid tumors are papillary carcinomas in origin [12-14]. Other types that have been reported are follicular, mixed follicular, medullary, papillary Hürthle cell, and anaplastic carcinoma [12, 15-17].

Radiological imaging, such as computed tomography (CT), Doppler US, and magnetic resonance imaging (MRI), may help in providing the location and extension of ectopic thyroid tissue, adding to a better pre-operative evaluation [18]. Confirming the diagnosis of ectopic thyroid is established by fine-needle aspiration cytology (FNAC), which is considered the most sensitive and specific diagnostic procedure to discriminate benign from malignant lesions in normal and ectopic thyroid pre-operatively [14]. Fine-needle aspiration cytology in some studies showed its accuracy in diagnosing submental masses with minimal false-positive results for diagnosis [19]. Despite that, FNAC might be non-diagnostic or misleading, especially in cystic lesions [14].

## Case presentation

A 48-year-old female patient, known case of hypothyroidism (on thyroxin 25 mcg) for 20 years, came to our clinic complaining of progressive, painless submental swelling for seven years. The patient did not experience dysphagia, odynophagia, dyspnea, hoarseness, stridor, aspiration, or fever. She denied any family history of thyroid cancer or previous radiation exposure. No symptoms of hyperthyroidism or hypothyroidism were reported.

Neck examination showed a 3 x 4 cm, firm, non-tender submental mass with no overlying skin abnormalities, no palpable lymph nodes. Fiber-optic nasopharyngoscopy showed bilateral mobile vocal folds. Other ENT examination was unremarkable. Thyroid function tests showed euthyroid levels. Other laboratory tests were within normal limits.

Thyroid ultrasound showed the absence of the thyroid gland in the pre-tracheal area.

**Figure 1:**
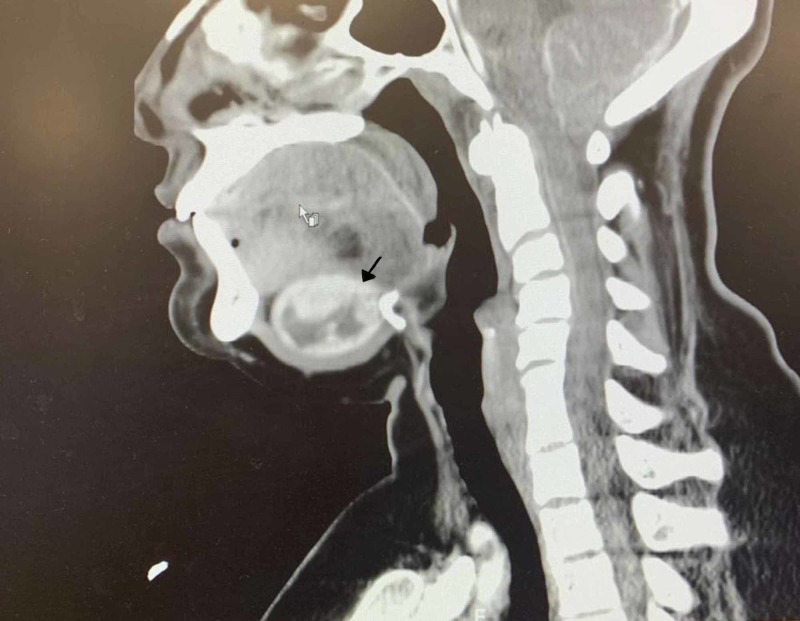
CT with contrast, lateral view demonstrating ectopic sub-lingual thyroid.

Neck CT (Figure 1) scan showed a 2.55 x 3.67 x 3.7 cm well capsulated sublingual mass, no invasion to the adjacent structures, no lymph nodes metastasis.

**Figure 2:**
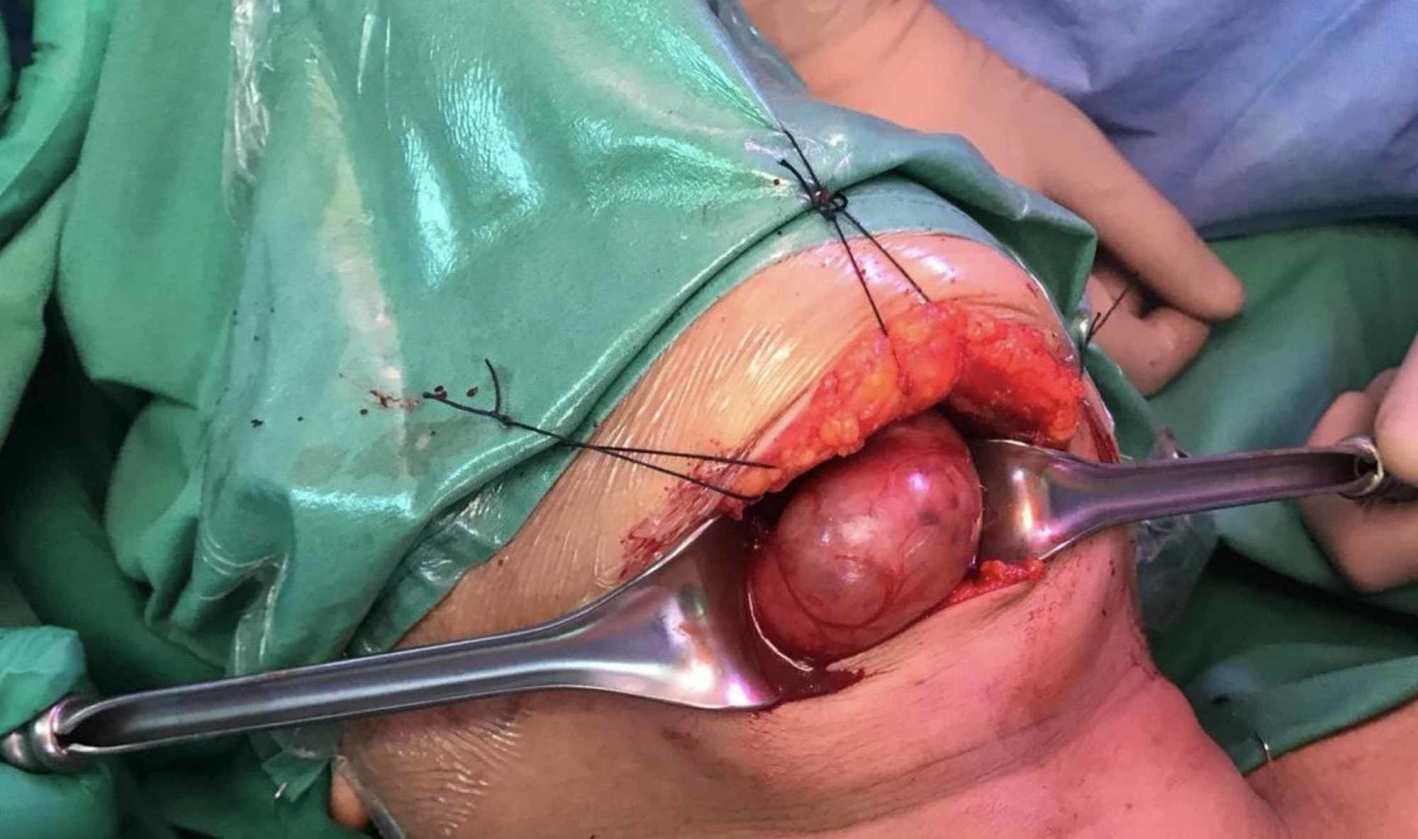
Intra-operative view of the sub-lingual thyroid.

**Figure 3:**
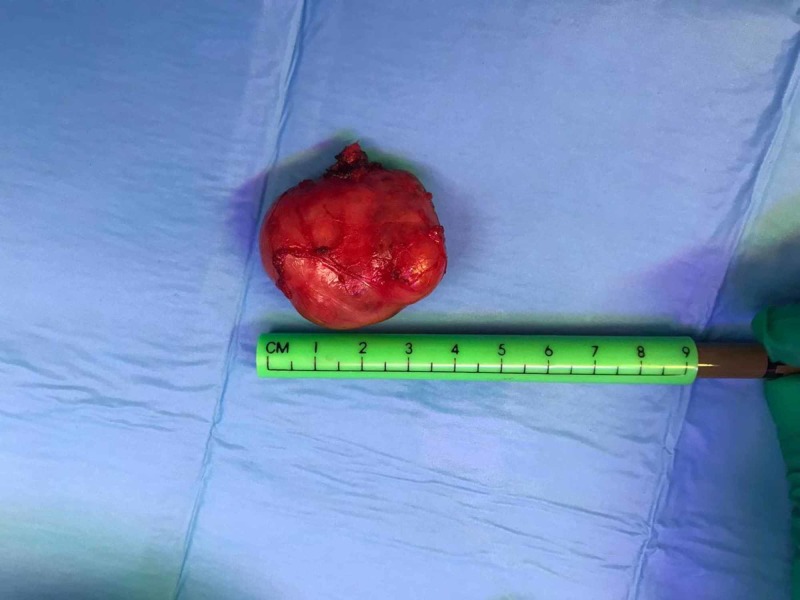
Sub-lingual thyroid after complete excision.

The patient underwent sublingual thyroid gland excision through an external transcervical submental approach (Figures 2-3), with excision of the mid-portion of the hyoid bone (as the tumor was attached to it), and a level 1A neck dissection. A drain was placed, and the patient was shifted to the Surgical Intensive Care Unit for observation. The patient was extubated and shifted to the surgical ward on day 1 postoperatively, the drain was removed on day 2 postoperatively, and the patient was discharged on the 3rd day postoperatively in a stable condition.

**Figure 4:**
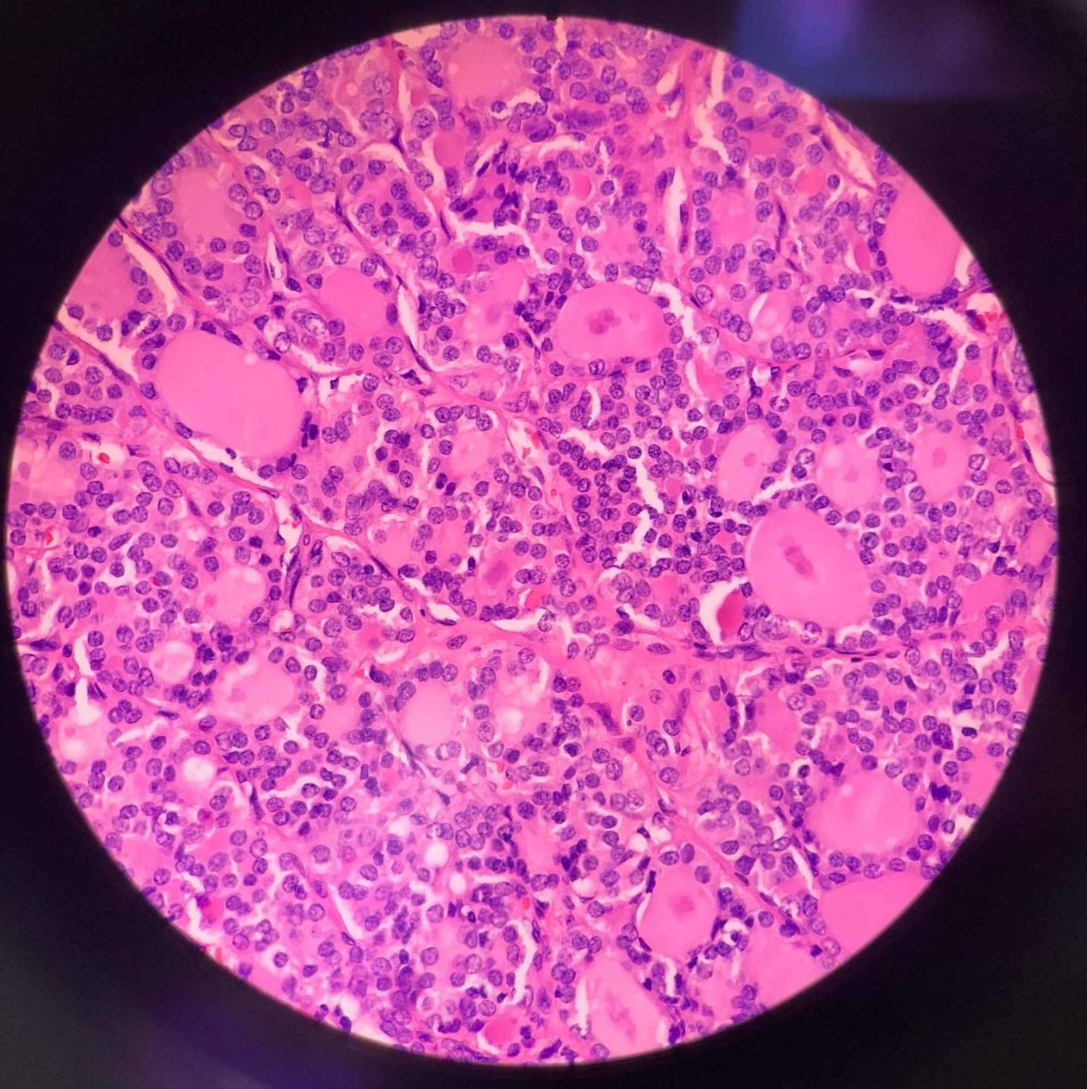
Microscopic image showing papillary microcarcinoma, follicular variant.

Histopathology report showed sublingual thyroid papillary microcarcinoma, 1.0 cm follicular variant with no extrathyroid invasion, and no pathological lymph nodes (Figure 4). Pathological staging T1aN0M0 was according to the TNM (tumor, node, metastasis) staging system. The case was discussed in the tumor board and planned for follow-up only, as radioactive iodine treatment was not indicated.

The patient was seen in the clinic after one week, one month, and three months' intervals, and was found to be completely asymptomatic with no complaint.

## Discussion

Ectopic thyroid is a rare clinical condition with prevalence ranging between 1 per 100,000 to 300,000 persons [1]. Female presentation accounts for the majority of the cases, with 65-80% in agreement with our case [5]. The mechanisms of thyroid dysgenesis are not fully understood. The studies have shown that a certain gene mutation like PAX8, TITF1 (NKX2-1), and FOXE1 (TITF2) may involve in developing this condition [2-4].

Ectopic thyroid could be located anywhere along its normal pathway. It could be lingual thyroid, sublingual thyroid, or around the hyoid bone as well as mediastinum or in the subdiaphragmatic area [5, 9]. In this case, the patient had a sub-lingual thyroid, which is considered a rare presentation of the ectopic thyroid gland.

The malignant shift is relatively rare, with a liability of carcinoma transformation in such tissue is below 1%. In earlier studies, follicular carcinomas were more prominent compared to papillary carcinomas [5]. Most recent studies suggested that papillary carcinoma, a follicular variant, has been increasingly recognized as becoming the most common type of malignancy in ectopic thyroid [5, 6]. In previous studies, around 100 cases of papillary carcinoma in ET have been addressed [14, 18]. In our case, the histopathology showed papillary microcarcinoma, follicular variant. Papillary thyroid microcarcinoma is a papillary carcinoma that is defined as papillary cancer with a diameter equal to or less than 10-15 mm.

The treatment approach for ectopic thyroid relies on many factors, such as patient’s age, symptoms, localization, malignancy, thyroid functional status, surgical, and anesthesiological risk [7]. Surgical management of ectopic thyroid is considered with obstructive symptoms, bleeding, or malignancy [8]. The surgical approach is preferred, as in this case, our patient was successfully treated with a transcervical/submental approach through a transverse incision. The mass was removed with the mid-portion of the hyoid bone (as in Sistrunk procedure), as the mass was attached to the hyoid bone. However, it might also decrease the recurrence rate, much like in thyroglossal duct cyst [20]. Other approaches mentioned in the literature were transoral, transcervical with a midline vertical incision, and submandibular approach. This patient was approached in transcervical/submental technique over transoral/sublingual for many reasons such as the large size of the mass (4 cm), avoiding the risk of injury to important structures (e.g., submental and sublingual salivary glands, lingual artery and lingual nerve), and for better swallowing and speaking outcome.

## Conclusions

Ectopic thyroid is a rare manifestation of thyroid diseases, diagnosing a sublingual/submental thyroid gland could be difficult, as it might be misdiagnosed as functional thyroid disease. It is four times more common in females than in males. Malignant transformation of the ectopic thyroid gland is considered a rare manifestation. In previous studies, follicular cancer of the ectopic thyroid gland was the most frequent type of cancer. However, such studies in recent years reported that papillary cancer has become widely recognized. The surgical approach depends on several factors. Sistrunk procedure in such cases should be studied more as this patient will be followed up for possible recurrence. Case reports about ectopic thyroid cancer are limited. In such cases, little work has been done to build a standardized clinical approach for an ectopic thyroid tumor.
